# Actionable Gene Expression-Based Patient Stratification for Molecular Targeted Therapy in Hepatocellular Carcinoma

**DOI:** 10.1371/journal.pone.0064260

**Published:** 2013-06-13

**Authors:** Jung-Hee Kwon, Namgyu Lee, Jin Young Park, Yun Suk Yu, Jin Pyo Kim, Ji Hye Shin, Dong-Sik Kim, Jae Won Joh, Dae Shick Kim, Kwan Yong Choi, Koo-Jeong Kang, Gundo Kim, Young Ho Moon, Hee Jung Wang

**Affiliations:** 1 Cbs Bioscience Inc., Daejeon, Korea; 2 Department of Life Sciences, Pohang University of Science and Technology, Pohang, Korea; 3 Division of HBP Surgery & Liver Transplantation, Department of Surgery, Korea University College of Medicine, Seoul, Korea; 4 Department of Surgery, Samsung Medical Center, Sungkyunkwan University School of Medicine, Seoul, Korea; 5 Department of Pathology, Samsung Medical Center, Sungkyunkwan University School of Medicine, Seoul, Korea; 6 Department of Surgery, Keimyung University School of Medicine, Daegu, Korea; 7 Department of Microbiology, Pukyong National University, Busan, Korea; 8 Department of Surgery, Ajou University School of Medicine, Suwon, Korea; The University of Hong Kong, China

## Abstract

**Background:**

The effectiveness of molecular targeted agents is modest in hepatocellular carcinoma (HCC). Efficacy of molecular targeted therapies has been better in cancer patients with high expression of actionable molecules defined as cognate target molecules. However, patient stratification based on the actionable molecules dictating the effectiveness of targeted drugs has remained understudied in HCC.

**Experimental Design & Results:**

Paired tumor and non-tumoral tissues derived from a total of 130 HCC patients were studied. Real-time RT-PCR was used to analyze the mRNA expression of actionable molecules in the tissues. mRNA levels of EGFR, VEGFR2, PDGFRβ, FGFR1, and mTOR were up-regulated in tumors compared to non-tumors in 35.4, 42.3, 61.5, 24.6, and 50.0% of patients, respectively. Up-regulation of EGFR was observed at early stage and tended to gradually decrease toward late stages (BCLC stage A: 41.9%; B: 30.8%; C: 17.6%). Frequency of VEGFR2 expression in tumors at stage C was lower than that in the other stages (BCLC stage A: 45.9%; B: 41.0%; C: 29.4%). PDGFRβ and mTOR were observed to be up-regulated in more than 50% of tumors in all the stages whereas FGFR1 was up-regulated in only about 20% of HCC irrespective of stages. A cluster analysis of actionable gene expression revealed that HCC can be categorized into different subtypes that predict the effectiveness of molecular targeted agents and combination therapies in clinical trials. Analysis of *in vitro* sensitivity to sorafenib demonstrated that HCC cells with up-regulation of PDGFRβ and c-Raf mRNA are more susceptible to sorafenib treatment in a dose and time-dependent manner than cells with low expression of the genes.

**Conclusions:**

mRNA expression analysis of actionable molecules could provide the rationale for new companion diagnostics-based therapeutic strategies in the treatment of HCC.

## Introduction

Hepatocellular carcinoma (HCC) is the most common primary liver malignancy. HCC is the sixth most common cancer and the third most common cause of cancer mortality in the world [1]. Many molecular targeted drugs have entered clinical trials as palliative and adjuvant treatments for HCC [2]. The multikinase inhibitor sorafenib was approved for the first-line therapy in advanced HCC as a result of a statistically significant but modest improvement of overall survival and time to progression in two randomized controlled trials [3], [4]. Other molecular targeted drugs have been tested in combination with sorafenib or in the adjuvant setting [5], [6]. However, the limited benefits of current molecular targeted agents including sorafenib have raised an urgent need for new therapeutic strategies in HCC [7].

Predictive biomarkers are increasingly used for diagnosis, prognosis, and therapeutic decision making in diverse cancers, propelling a paradigm shift in the management of cancer [8]–[10]. Biomarkers have helped to stratify patients and thus achieve better outcomes from a given drug in the clinic. Trastuzumab, a HER2 targeting monoclonal antibody, is effective in metastatic breast cancer patients with 3+ HER2 overexpression assessed by IHC or HER2 gene amplification [11]. Also, patients with non-small cell lung cancer harboring activating mutations within the kinase domain of EGFR show impressive clinical responses to the EGFR inhibitor gefitinib [12]. This type of molecular classification which stratifies individual tumors into molecular subtypes for which targeted therapy could have potential efficacy is described as actionable molecular subtyping [13], [14].

In the current study, we aimed to stratify HCC patients by cluster analysis of mRNA expression of EGFR, VEGFR2, PDGFRβ, FGFR1, and mTOR, which are major targets for molecular targeted therapy, as actionable molecules. An *in vitro* sorafenib sensitivity test demonstrated that actionable gene expression-based molecular subtyping may maximize the efficacy of molecular targeted therapy. Our results could provide novel insights for the strategic design of effective molecular targeted treatment in HCC.

## Patients, Materials and Methods

### Patients and tissue samples

HCC tissues and corresponding non-cancerous hepatic tissues were obtained with informed consent from 130 patients who had undergone curative resection for primary HCC between 1998 and 2006 at the Ajou and Samsung Medical Centers in South Korea. The study protocol was approved by the Institutional Review Board of each Medical Center. Table 1 summarizes the demographic characteristics of the 130 HCC patients investigated in the current study. We used BCLC stage and Edmondson and Steiner grade according to the published criteria [15], [16].

**Table 1:** Demographic characteristics of HCC patients.

Variables			Variables		
Age	<55 years	80	Child-Pugh class	A	126
	≥55 years	50		B	4
Gender	Male	97		C	0
	Female	33	BCLC stage	A	74
HBV	Absent	30		B	39
	Present	100		C	17
HCV	Absent	118	Vascular invasion	Absent	57
	Present	12		Present	73
Liver cirrhosis (−1)*	Absent	69	Tumor number	Single	104
	Present	60		Muliple	26
AFP level	<100 ng/mL	75	Tumor size	≤5 cm	93
	≥100 ng/mL	55		>5 cm	37
Tumor stage	I	55	Edmondson grade	I	18
	II	49		II	95
	III	25		III	17
	IV	1		IV	0

* minus values mean the number of patients without the relevant clinicopahologic information; HBV: Hepatitis B Virus; HCV: Hepatitis C Virus; AFP: Alpha fetoprotein; BCLC: The Barcelona Clinic Liver Cancer.

### Real-time RT-PCR

Real-time RT-PCR was carried out as described previously [17]. The primers and probes were labeled with FAM and TAMRA at the 5′ end and 3′ end, respectively (Table S1). The mRNA levels of EGFR, VEGFR2, PDGFRβ, FGFR1, and mTOR were measured (the threshold cycle, Ct value) in triplicate and then normalized to a set of reference genes (B2M, GAPDH, HMBS, HPRT1, and SDHA) by subtracting the average values of the mRNA levels of the reference genes as an internal control [18]. The mRNA copy number ratios were calculated as 2^−ΔCt (^ΔCt values  =  target gene Ct – average Ct of reference genes).

### Cell culture and viability assay

SK-Hep1, Hep3B, HepG2 and Huh-7 HCC cell lines were obtained from the Korean Cell Line Bank (Korea). The cells were cultured in DMEM supplemented with 10% FBS, 100 U/ml of penicillin and 100 μg/ml streptomycin in a 37°C humidified incubator conditioned with 5% CO_2_.

The effect of sorafenib (Santa Cruz Biotechnology) on cell viability was assessed using the WST-1 reagent (Roche). Cells were exposed to the indicated concentrations of sorafenib in DMEM with 5% FBS for 24 hours. The effect of sorafenib on cell viability was evaluated at the indicated time in media containing 2.5 μM of sorafenib. After sorafenib treatment for the indicated time, the culture media was exchanged with WST-1 containing media (1:10 final dilution). Following additional incubation of the cells for 2 hours, absorbance at 450 nm was measured using a microplate reader (Spectrafluor Plus, Tecan).

### Statistical analysis

Statistical analyses in this study were carried out with the open source statistical programming environment R. 2^−ΔCt^ values of each gene were shown in box and whisker plot and the difference between tumor and non-tumor tissues was evaluated for significance using Student's t test. The relationship of gene expression with clinicopathologic variables was evaluated using χ2 and Fisher's exact tests. Up-regulated, unchanged, or down-regulated gene expression was evaluated using the fold difference of 2^−ΔCt^ values between the tumors and the surrounding non-tumoral tissues. Hierarchical clustering analyses of the T/N ratios (tumor/non-tumor) of 2^−ΔCt^ values were performed for patient stratification. For the cell viability results, a two-tailed t test was used and P values of <0.05 were considered statistically significant (marked with an asterisk).

## Results

### Expression of actionable molecules in the paired tissues of tumor and surrounding normal liver

In many kinds of cancer, the expression of actionable molecules has been shown to be aberrantly regulated. We therefore investigated mRNA expression for 5 actionable molecules (EGFR, VEGFR2, PDGFRβ, FGFR1, and mTOR) in 130 HCC and matched non-tumoral tissues.

**Figure 1:**
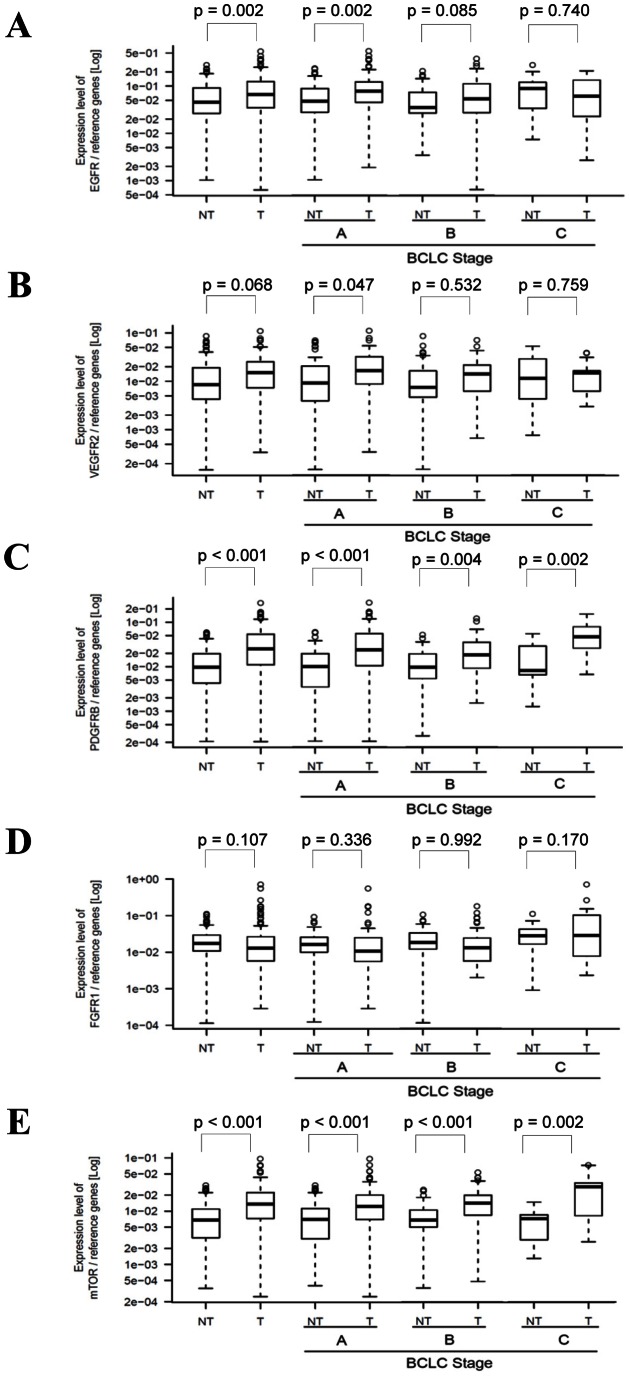
mRNA expression of actionable molecules in HCC patients. Box and whisker plot for expression of EGFR (A), VEGFR2 (B), PDGFRβ (C), FGFR1 (D), and mTOR (E) in non-cancerous hepatic (NT) and matched tumor (T) tissues determined by real-time RT-PCR. The box is marked by the first and third quartile with the median marked by a thick line. The whiskers extend to the most extreme data point which is no more than 1.5 times the interquartile range from the box. A, B, and C: BCLC stage A, B, and C, respectively.

The average mRNA levels of EGFR were significantly higher in the tumors than in non-cancerous hepatic tissues (1.47 fold difference in mean copy number ratios, P = 0.002) (Fig. 1A). EGFR mRNA expression was increased in tumors diagnosed at BCLC stage A (1.64 fold, P = 0.002) and this expression pattern was maintained in BCLC stage B tumors (Fig. 1A). The mean expression of VEGFR2 was up-regulated in all tumors, but the expression difference was statistically significant only for BCLC stage A tumors (1.38 fold, P = 0.047; Fig. 1B). PDGFRβ mRNA was significantly overexpressed in all the tumors irrespective of BCLC stage (2.93, 3.29, 2.02, 3.41 fold in all tumors and BCLC stages A, B, and C, respectively; Fig. 1C). The differences in the FGFR1 mRNA levels between tumors and non-tumoral tissues were not statistically significant (Fig. 1D). Up-regulation of mTOR also occurred in tumor tissues and was independent of BCLC stage (2.15, 1.89, 2.05, 3.88 fold in total, BCLC stages A, B, and C, respectively; Fig. 1E).

### Characterization of the frequency and stage of actionable gene expression

**Figure 2:**
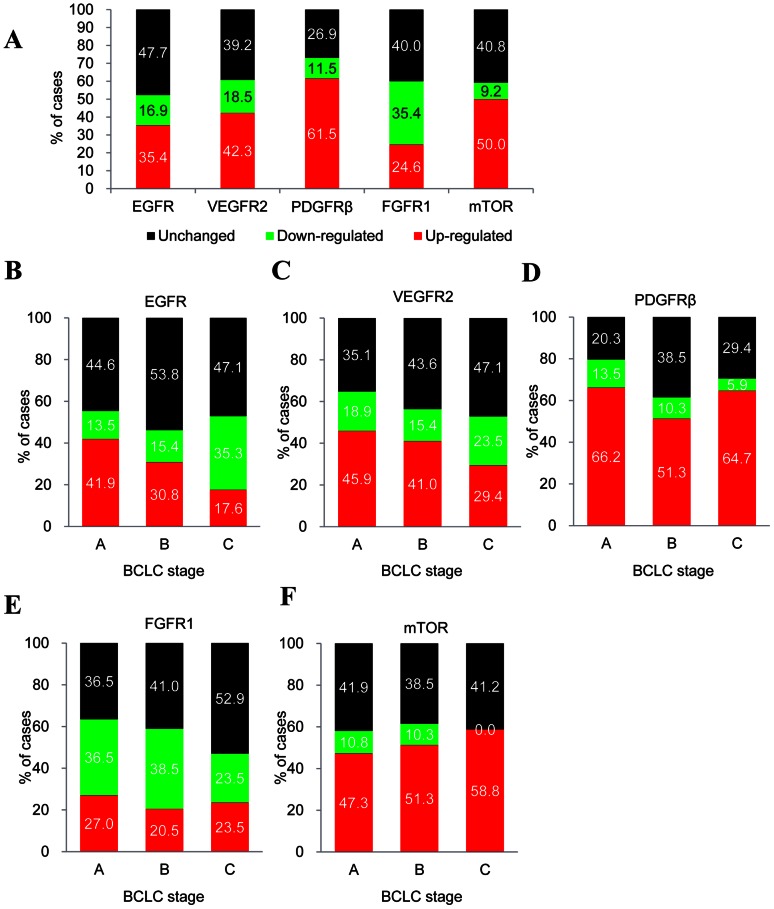
Expression frequency and timing of actionable molecules in HCC patients. (A) mRNA expression frequency of EGFR, VEGFR2, PDGFRβ, FGFR1, and mTOR in tumors; (B–F) Expression timing for EGFR, VEGFR2, PDGFRβ, FGFR1, and mTOR. Up-regulated: increased expression more than 2.0-fold in tumors than in non-tumors; down-regulated: reduced expression more than 2.0-fold in tumors than in non-tumors; unchanged: gene expression within −2.0∼2.0 fold in tumors than in non-tumors. The data are presented in % of cases.

Since overexpression is one of the mechanisms underlying activation of actionable molecules in cancers, we investigated the frequency of actionable gene overexpression in tumors. EGFR mRNA levels were up-regulated in 35.4% of the tumors and unchanged in 47.7% of the tumors. VEGFR2 was up-regulated, unchanged, and down-regulated in 42.3%, 39.2%, and 18.5% of the tumors, respectively. PDGFRβ was up-regulated in tumors at a high rate (61.5%). While patients with unchanged and down-regulated expression of FGFR1 were 40% and 35.4%, respectively, only 24.6% of the patients showed up-regulation of FGFR1 in the tumors. mTOR was found to be up-regulated in half of the tumors (Fig. 2A).

We next examined the association of expression of actionable molecules with HCC stage across the different BCLC stages. Up-regulation of EGFR was observed in 41.9% of the tumors at BCLC stage A but the proportion decreased by 17.6% at later stages and down-regulation of EGFR was prominently found in advanced stage tumors (35.3%; Fig. 2B). The stage association of VEGFR2 up-regulation was similar to that of EGFR (Fig. 2C). Up-regulation of PDGFRβ was observed in 66.2%, 51.3%, and 64.7% of the tumors at BCLC stages A, B, and C, respectively (Fig. 2D). FGFR1 levels were up-regulated in about 20% of the tumors irrespective of stage (Fig. 2E). mTOR was up-regulated in about half of the tumors in all the stages (Fig. 2F).

### Association of clinical characteristics with actionable gene expression

To gain further insight into the gene expression of actionable molecules in HCC, the relationships between mRNA levels of actionable genes and clinicopathologic features were investigated. High mRNA expression of EGFR was correlated with the BCLC stage (P = 0.049, Table 2). The degree of tumor differentiation (Edmondson grade) was significantly associated with expression of both EGFR and VEGFR2 (P = 0.003 and 0.004, respectively), which showed that well-differentiated HCC tended to express EGFR and VEGFR2 at high levels. EGFR was significantly overexpressed in single tumors (P = 0.001).

**Table 2:** Relationships between mRNA expression of actionable molecules and clinicopathologic features.

		EGFR			VEGFR2			PDGFRβ			FGFR1			mTOR		
		Low (n = 22)	High (n = 46)	p Value	Low (n = 24)	High (n = 55)	p Value	Low (n = 15)	High (n = 80)	p Value	Low (n = 46)	High (n = 32)	p Value	Low (n = 12)	High (n = 65)	p Value
Age	<55 years	15	24	0.324	18	30	0.144	9	51	0.988	26	24	0.152	8	41	1
	≥55 years	7	22		6	25		6	29		20	8		4	24	
Gender	Male	17	33	0.849	16	40	0.783	10	60	0.53	35	21	0.451	9	44	0.744
	Female	5	13		8	15		5	20		11	11		3	21	
HBV	Absent	4	14	0.437	3	18	0.111	2	21	0.348	7	3	0.513	0	13	0.201
	Present	18	32		21	37		13	59		39	29		12	52	
HCV	Absent	19	41	0.707	22	47	0.715	14	72	1	41	31	0.392	12	60	1
	Present	3	5		2	8		1	8		5	1		0	5	
Liver cirrhosis	Absent	12	26	0.991	14	33	0.985	7	46	0.587	21	18	0.403	5	34	0.717
	Present	10	19		10	21		8	33		25	13		7	31	
Tumor stage	I-II	15	40	0.098	18	43	0.985	14	64	0.293	40	26	0.536	12	50	0.109
	III-IV	7	6		6	12		1	16		6	6		0	15	
Child-Pugh class	A	22	44	1	22	53	0.581	15	77	1	45	32	1	12	64	1
	B	0	2		2	2		0	3		1	0		0	1	
BCLC stage	A	10	31	**0.049**	14	34	0.614	10	49	0.844	27	20	0.7	8	35	0.456
	B	6	12		6	16		4	20		15	8		4	20	
	C	6	3		4	5		1	11		4	4		0	10	
AFP level	<100 ng/ml	10	29	0.267	11	39	0.061	9	49	0.844	24	21	0.342	7	39	1
	≥100 ng/ml	12	17		13	16		6	31		22	11		5	26	
Vascular invasion	Absent	7	22	0.324	7	29	0.091	8	34	0.623	17	14	0.713	6	28	0.899
	Present	15	24		17	26		7	46		29	18		6	37	
Tumor number	Single	11	41	**0.001**	18	47	0.338	13	67	1	38	26	0.884	11	52	0.684
	Muliple	11	5		6	8		2	13		8	6		1	13	
Tumor size	≤5 cm	15	35	0.691	17	40	0.92	11	61	0.754	32	24	0.788	9	46	1
	>5 cm	7	11		7	15		4	19		14	8		3	19	
Edmondson grade	I	0	14	**0.003**	0	15	**0.004**	2	12	1	4	6	0.302	1	12	0.679
	II-III	22	32		24	40		13	68		42	26		11	53	

### Patient subtyping by clustering analysis of actionable gene expression

As molecular targeted therapy could be guided by the molecular subtypes reflecting the status of actionable molecules in individual tumors, we carried out a hierarchical clustering analysis to specify the patients harboring high levels of actionable genes.

**Figure 3:**
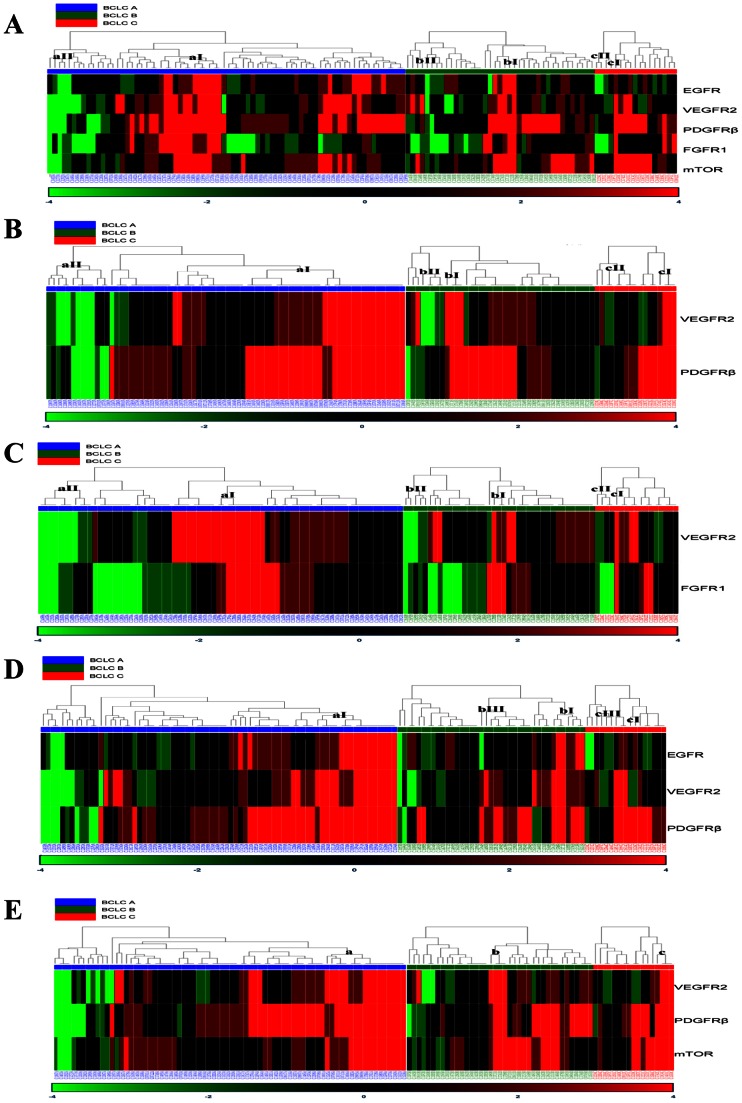
Patient stratification by hierarchical clustering analysis of actionable gene expression. Each row and column means an individual gene and an individual patient sample, respectively. After categorization by BCLC stages, the patients were ordered by Euclidean distance and linkage according to the ratios of 2^−ÄCt^ values in each tumor compared to the cognate non-tumor. In the heat map, the red and green color reflect high and low expression levels, respectively, as depicted in the scale bar at the bottom. The scale represents the gene expression ratios from 4 to −4 in fold difference of 2^−ÄCt^ values. (A) Hierarchical clustering of 5 actionable genes in HCC patients. (B) Hierarchical clustering of VEGFR2 and PDGFRβ. (C) Hierarchical clustering of VEGFR2 and FGFR1. (D) Hierarchical clustering of VEGFR2, PDGFRβ, and EGFR. (E) Hierarchical clustering of VEGFR2, PDGFRβ, and mTOR.

Hierarchical cluster analysis revealed that the common expression patterns of EGFR, VEGFR2, PDGFRβ, FGFR1, and mTOR genes did not fall into distinct patient clusters significantly associated with BCLC stages (Fig. S1). After categorization of BCLC stages, the hierarchical cluster analysis was performed to specifically classify the patients into subtypes according to the mRNA expression of actionable molecules. Patients with expression ratios of EGFR, VEGFR2, PDGFRβ, FGFR1, and mTOR at least twofold higher in tumors relative to non-tumors were clustered in subtype I at BCLC stages A, B, and C, respectively. In contrast, subtype II tumors that expressed all the genes at low levels were clustered in subtype II (Fig. 3A).

Since molecular targeted agents have multiple targets, molecular subtyping could not provide enough information for effectiveness of the drugs until it utilizes various markers. Tumors with up-regulation of sorafenib-targets VEGFR2 and PDGFRβ, which are hypothetically amenable to sorafenib, were classified in subtype I across the BCLC stages and the relative proportions of patients in each subtype gradually decreased toward BCLC stage C (a: 40.54%, b: 30.77%, c: 23.53%; Fig. 3B). In a, b, and c subgroups, the proportions of patients highly expressing VEGFR2 and FGFR1, who are expected to benefit from molecular targeted agents against VEGFR2 and FGFR1 including brivanib, were 22.97%, 10.26%, 5.88%, respectively (Fig. 3C).

In the view of cross-talk between molecular targeted pathways and combination therapy under current clinical trials, VEGFR2 and PDGFRβ as sorafenib-target genes and other actionable genes were analyzed. Patients in subtype III at each stage exhibited down-regulation of EGFR and up-regulation of VEGFR2 and PDGFRβ. In addition, patients who overexpressed all the genes were classified in subtype I (Fig. 3D). Subtypes a, b, and c displayed up-regulation of VEGFR2 and PDGFRβ with mTOR for combined treatment of sorafenib and mTOR inhibitor (Fig. 3E).

### Sorafenib sensitivity according to expression of sorafenib-target molecules in HCC cell lines

**Figure 4:**
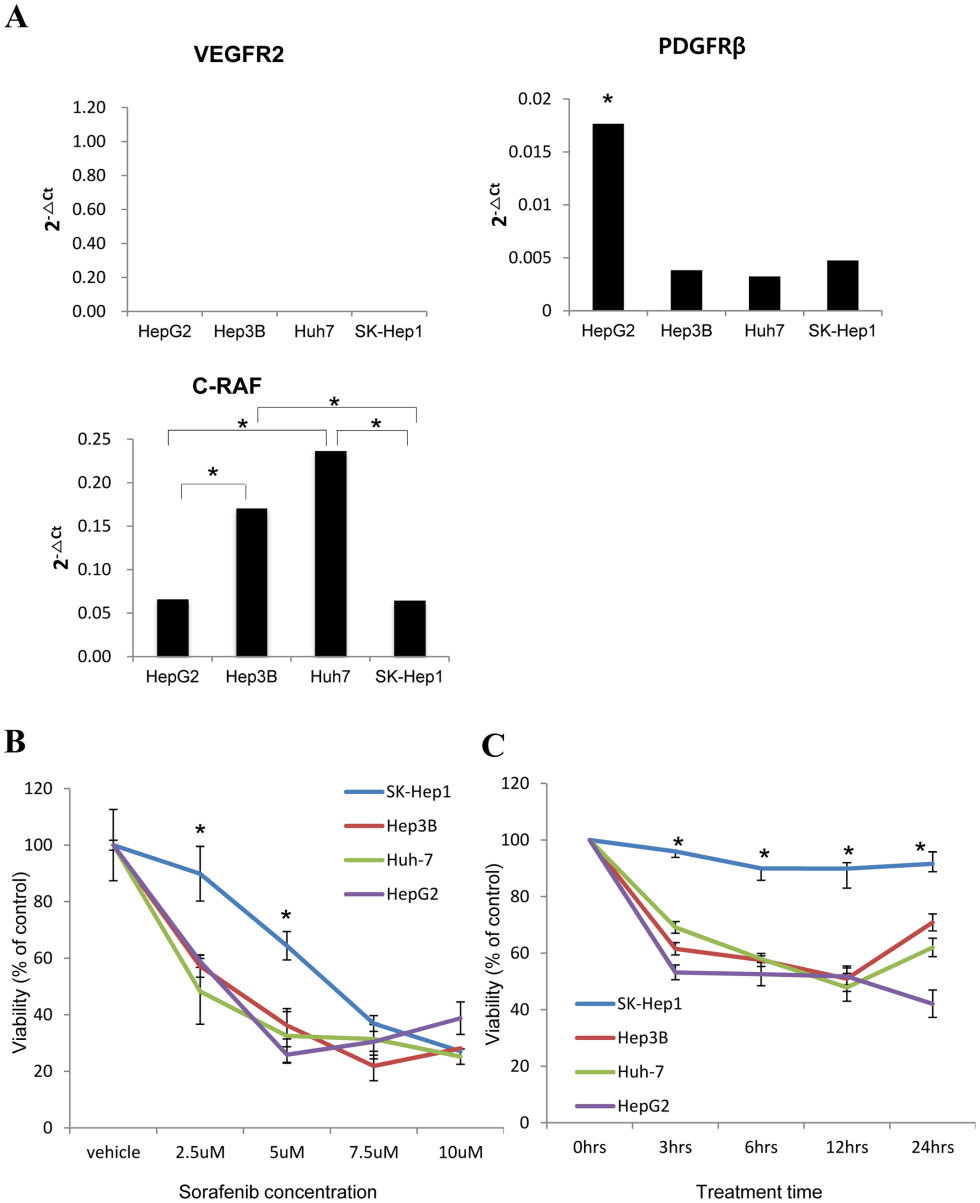
Analysis of mRNA expression of sorafenib-target genes and sorafenib sensitivity in HCC cell lines. (A) To examine the mRNA levels of VEGFR2, PDGFRβ, and c-Raf, real-time quantitative RT-PCR was performed with cDNA derived from SK-Hep-1, Hep3B, Huh-7 and HepG2 cell lines. Expression of the genes was plotted in bar graphs using 2^−ΔCt^ values of each gene and the significant difference in gene expression among the cells were marked with asterisk (*). (B, C) The susceptibilities of HCC cell lines to sorafenib treatment were evaluated by WST-1 cell viability assay. The significant differences between SK-Hep-1 and the other cells were marked with asterisk (*). (B) SK-Hep-1, Hep3B, Huh-7 and HepG2 cells were incubated for 24 hours at the indicated concentrations of sorafenib. (C) SK-Hep-1, Hep3B, Huh-7 and HepG2 cells were treated with 2.5 μM of sorafenib for the indicated time. After incubation in sorafenib-containing media, WST-1 was added to the cells for 2 hours and then the cell viabilities were measured. The final cell survival (%) values were calculated relative to the value of vehicle and at 0 hours in concentration and time-dependent data, respectively.

To verify whether the mRNA expression of targeted molecules is associated with the effectiveness of molecular targeted drugs, mRNA expression of sorafenib-target genes VEGFR, PDGFRβ, and c-Raf and *in vitro* sorafenib sensitivity were investigated in HCC cell lines. VEGFR2 was not expressed in any of the cell lines. While PDGFRβ mRNA was highly expressed in HepG2, there was relatively little expression in the other cell lines. Hep3B and Huh7, with the lowest expression of PDGFRβ, exhibited significantly higher expression of c-Raf compared to HepG2 and SK-Hep-1. HepG2, which expressed PDGFRβ mRNA at a high level, showed relatively low mRNA expression of c-Raf (Fig. 4A). Whether the different mRNA levels of sorafenib-target molecules could affect the sensitivity of the HCC cells to sorafenib was examined. SK-Hep-1, the cell lines with the lowest levels of both PDGFRβ and c-Raf, was the most resistant to sorafenib at concentrations less than 5 μM. On the contrary to SK-Hep-1 cells, HepG2, Huh-7, and Hep3B with overexpression of PDGFRβ or c-Raf were more susceptible to the same concentration of sorafenib (Fig. 4B). These results were consistent with the cell viability results when cells were treated with 2.5 μM sorafenib in a time-dependent manner (Fig. 4C).

## Discussion

Because of tumor selectivity and low toxicity, molecular targeted therapy is considered ideal for cancer patients. Since sorafenib was approved as the first-line treatment for advanced HCC, a number of molecular targeted drugs have been studied in clinical trials. However, the efficacy of targeted agents including sorafenib has proven to be modest and second-line therapies in combination with sorafenib have shown disappointing results [7], [19].

Actionable molecular subtyping has been implemented for patient stratification in some types of cancer. Lung cancer has nine subtypes based on specific genetic aberrations including overexpression and mutations [13] and melanoma is categorized into eight subtypes associated with targeted treatment [14]. Inspired by these approaches, we investigated for the first time expression of five major actionable molecules, EGFR, VEGFR2, PDGFRβ, FGFR1, and mTOR in 130 HCC patients. Because measuring mRNA expression is more quantitative and reproducible, and feasible to implement as a multiplex analysis compared to measuring protein expression using methods such as IHC, and therefore real-time quantitative RT-PCR was used in this study. mRNA levels of EGFR were significantly higher in tumor tissues than in non-tumor tissues (Fig. 1A) and the overexpression rate was 35.4% (Fig. 2A). Our result is consistent with several prior studies that have demonstrated that EGFR is overexpressed in HCC [20]. Additionally, VEGFR2 and PDGFRβ were also highly expressed in HCC tumors (Fig. 1B and C), in accordance with previous reports [21], [22]. Up-regulation of mTOR was more pronounced with the progression of HCC (Fig. 2F), which agrees with the results of Zhou, L et al [23]. Notably, a considerable number of tumors at BCLC stages A and B overexpressed the actionable molecules implying the applicability of molecular targeted treatment at an earlier stage in HCC. Taken together, our results demonstrating the overexpression of actionable molecules in early stages in addition to the advanced stage of HCC suggest that molecular targeted treatment at an earlier stage, along with adjuvant therapy, may be a new effective treatment strategy.

Traditionally, therapy decisions in HCC have been made according to the histologic features of tumors. However, due to the limitations of conventional classification systems in treatment decisions, molecular classes in HCC have been suggested by several research groups. The Mount Sinai-Barcelona-Milan-Broad Institute groups have proposed IGF-Akt-mTOR, Wnt-β-catenin, and IFN signaling pathways as major molecular classes in HCC [19]. Using global gene expression profiling, prognosis-associated genes have been categorized into subsets of HCC [24] and suggested as a classifier through a risk scoring method [25]. These approaches have characterized molecular subtypes of HCC but their validity in predicting the effectiveness of anti-cancer drugs has not yet been proven.

Actionable molecules have been recently utilized to classify HCC patients. VEGFR family and PDGFRα and β have been assessed as biomarkers to select HCC patients for targeted therapy by immunohistochemistry and tissue microarray [21], [26]. Plasma biomarkers including VEGF were analyzed in SHARP trials [27]. Taking a different approach from previous studies, we stratified HCC with mRNA expression of actionable molecules directly associated with the targeted drug mechanism of action. The clustering method classified patients into subtypes with overexpression, down-regulation, and unchanged expression of actionable genes (Fig. 3). Since the efficacy of molecular targeted therapies has been better in patients with high expression of cognate molecules [11], [28], patients in subtype aI, bI, and cI that highly express a set of VEGFR2-PDGFRβ or VEGFR2-FGFR1 may be susceptible to sorafenib or brivanib treatment, respectively. In contrast, subtypes aII, bII, and cII which had higher expression of the cognate actionable molecules in non-tumor rather than tumor tissues, would be more likely to have side effects without anti-tumor efficacy (Fig. 3B and C). To validate this supposition, we evaluated the responsiveness to sorafenib of human HCC cell lines showing differential expression of sorafenib-target genes. The complete absence of VEGFR2 expression in all HCC cell lines (Fig. 4A) is in line with previous reports showing primary expression of VEGFR2 in vasculature rather than tumor cells [29], [30]. As expected, SK-Hep-1 having the lowest mRNA levels in both PDGFRβ and c-Raf (Fig. 4A) was the most resistant to sorafenib treatment in both a dose- and time-dependent manner (Fig. 4B and C). In contrast, Hep3B and Huh7, who had a PDGFRβ mRNA expression level as low as SK-Hep-1 but high expression of c-Raf, another sorafenib target molecule (Fig. 4A), are both sensitive to sorafenib (Fig. 4B and C). These results clearly demonstrated that overexpression of either sorafenib-target gene confers susceptibility to sorafenib treatment in HCC. Taken together, our results suggest that actionable molecular subtyping by mRNA expression analysis could be a potent strategy for stratification of HCC patients amenable to molecular targeted therapy.

Low responsiveness to sorafenib in HCC could be ascribed to resistance mechanisms separate from those attributable to low expression of the molecular targets of sorafenib. Although mutation in drug target genes is one of the major causes for drug resistance in many types of cancer, mutations in actionable molecules have been uncommon in HCC, for example, EGFR activating mutations have been reported to occur in 0∼1% of HCC [31], [32] and Raf mutations have never been described in HCC [33]. The EGFR pathway is one of the mechanisms underlying sorafenib resistance. An *in vitro* study revealed that EGFR pathways are activated in HCC cells with resistance to sorafenib [34], [35]. These cells are more susceptible to sorafenib treatment when it is combined with the EGFR inhibitor gefitinib than when treated with sorafenib alone or gefitinib alone. Contrary to this result, another EGFR inhibitor, erlotinib, showed little anti-tumor activity and no synergistic effect upon co-treatment with sorafenib in a rat model of HCC [36]. To address the issue of combination therapy, we categorized HCCs according to the mRNA expression of molecules inhibited by combination therapy under clinical trials. Considering that EGFR activation causes sorafenib resistance, subtype III showing low expression of EGFR with high levels of VEGFR2 and PDGFRβ may be sensitive to sorafenib whereas subtype I with all the molecules up-regulated may be resistant to sorafenib treatment (Fig. 3D). Interestingly, mRNA expression of EGFR moderately correlated with those of VEGFR2 and PDGFRβ, supporting the potential effectiveness of combined treatment with an EGFR inhibitor and sorafenib as shown in previous reports [34], [35]. However, it should be determined in clinical trials whether up-regulation of EGFR is beneficial or not in sorafenib or combination treatments. Further clustering results indicated that most of the tumors overexpressing mTOR (subtypes a, b, and c) also highly expressed VEGFR2 and PDGFRβ (Fig. 3E), which strongly supports a potential synergistic effect of the mTOR inhibitors combined with sorafenib in HCC [37]–[39]. Additionally, this result implies that combined treatment with sorafenib plus mTOR inhibitor could help overcome both sorafenib and rapamycin resistance [38], [40]. Taken together, our findings provide molecular evidence that combined treatment of molecular targeted agents may be reasonable in HCC patients.

To our knowledge, we stratified for the first time HCC patients according to the mRNA expression of actionable molecules. Our findings provide the rationale for a new treatment strategy based on companion diagnostics toward molecular targeted therapy in HCC and warrant further investigation in prospective clinical trials.

## Supporting Information

Figure S1 **Hierarchical clustering analysis of 5 actionable genes.** Each row and column means an individual gene and an individual patient sample, respectively. the patients were ordered by Euclidean distance and linkage according to the ratios of 2^−ÄCt^ values in each tumor compared to the cognate no-tumor. In the heat map, the red and green color reflect high and low expression levels, respectively, as depicted in the scale bar at the bottom. The scale represents the gene expression ratios from 4 to −4 in fold difference of 2^−ÄCt^ values.
(TIF)

Table S1 **Sequences of primer and probes used in this study.**
(DOCX)
