# Supplementary figures and images for: Actionable Gene Expression-Based Patient Stratification for Molecular Targeted Therapy in Hepatocellular Carcinoma
Source: PLoS One. 2013 Jun 13;8(6):e64260. doi: 10.1371/journal.pone.0064260 (PMC3681850; doi:10.1371/journal.pone.0064260)

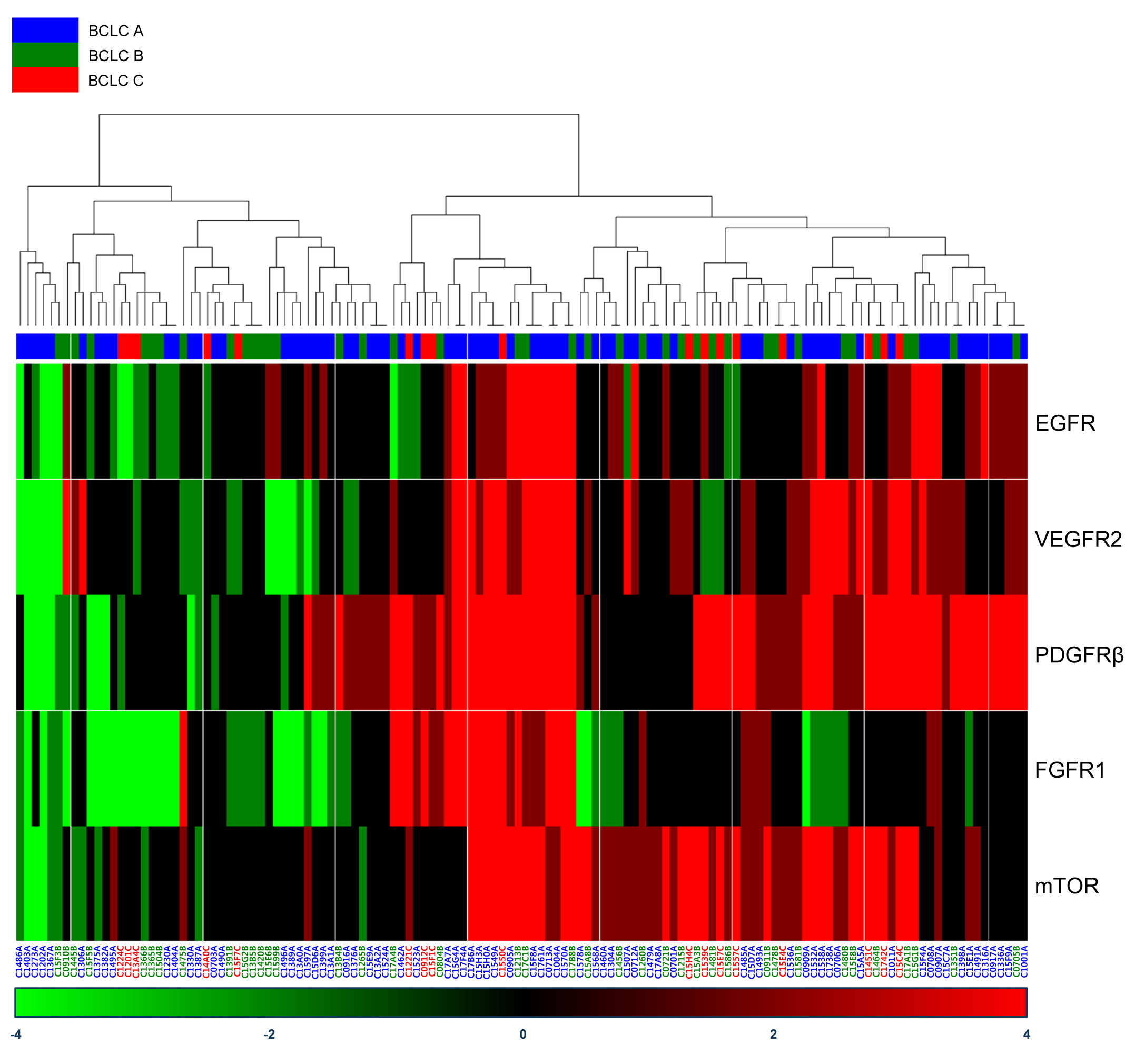

Supplement: Figure S1 — Hierarchical clustering analysis of 5 actionable genes. Each row and column means an individual gene and an individual patient sample, respectively. the patients were ordered by Euclidean distance and linkage according to the ratios of 2−ÄCt values in each tumor compared to the cognate no-tumor. In the heat map, the red and green color reflect high and low expression levels, respectively, as depicted in the scale bar at the bottom. The scale represents the gene expression ratios from 4 to −4 in fold difference of 2−ÄCt values. (TIF) [file pone.0064260.s001.tif]
