# Supplementary material for: Actionable Gene Expression-Based Patient Stratification for Molecular Targeted Therapy in Hepatocellular Carcinoma
Source: PLoS One. 2013 Jun 13;8(6):e64260. doi: 10.1371/journal.pone.0064260 (PMC3681850; doi:10.1371/journal.pone.0064260)
Supplement: Table S1 — Sequences of primer and probes used in this study. (DOCX) [file pone.0064260.s002.docx]

Table S1. Sequences of primer and probes used in this study

| Gene |  | sequence |
| --- | --- | --- |
| B2M | F | CATTCGGGCCGAGATGTCT |
|  | R | CTCCAGGCCAGAAAGAGAGAGTAG |
|  | P | CCGTGGCCTTAGCTGTGCTCGC |
| GAPDH | F | CACATGGCCTCCAAGGAGTAA |
|  | R | TGAGGGTCTCTCTCTTCCTCTTGT |
|  | P | CTGGACCACCAGCCCCAGCAAG |
| HMBS | F | CCAGGGATTTGCCTCACCTT |
|  | R | AAAGAGATGAAGCCCCCACAT |
|  | P | CCTTGATGACTGCCTTGCCTCCTCAG |
| HPRT1 | F | GCTCGAGATGTGATGAAGGAGAT |
|  | R | CCAGCAGGTCAGCAAAGAATT |
|  | P | CCATCACATTGTAGCCCTCTGTGTGCTC |
| SDHA | F | CACCTAGTGGCTGGGAGCTT |
|  | R | GCCCAGTTTTATCATCTCACAAGA |
|  | P | TGGCACTTACCTTTGTCCCTTGCTTCA |
| EGFR | F | GAAGGAGCTGCCCATGAGAA |
|  | R | GACTATGTCCCGCCACTGGAT |
|  | P | AAATCCTGCATGGCGCCGTGC |
| VEGFR2 | F | CACCACTCAAACGCTGACATGTA |
|  | R | CCAACTGCCAATACCAGTGGAT |
|  | P | TATGCCATTCCTCCCCCGCATCA |
| PDGFRβ | F | AGCGCTGGCGAAATCG |
|  | R | TTCACGCGAACCAGTGTCA |
|  | P | CTGTCCACGCGCAACGTGTCG |
| FGFR1 | F | CACGGGACATTCACCACATC |
|  | R | GGGTGCCATCCACTTCACA |
|  | P | ACTATAAAAAGACAACCAACGGCCGACTGC |
| mTOR | F | AGGCCGCATTGTCTCTATCAA |
|  | R | GCAGTAAATGCAGGTAGTCATCCA |
|  | P | TGCAATCCAGCTGTTTGGCGCC |
| C-RAF | F | GAGGTCGACATCCACACCTAATG |
|  | R | TCGAATTGCATCCTCAATCATC |
|  | P | CCACATGGTCAGCACCACCCTGC |
